# Socioeconomic factors differentiating maternal and child health-seeking behavior in rural Bangladesh: A cross-sectional analysis

**DOI:** 10.1186/1475-9276-9-9

**Published:** 2010-04-03

**Authors:** Ruhul Amin, Nirali M Shah, Stan Becker

**Affiliations:** 1 Department of Population, Family, and Reproductive Health, Johns Hopkins University, 615 N. Wolfe St, Baltimore, Md 21205, USA; 2 Department of International Health, Johns Hopkins University, 615 N. Wolfe St, Baltimore, Md 21205, USA

## Abstract

**Background:**

There has been an increasing availability and accessibility of modern health services in rural Bangladesh over the past decades. However, previous studies on the socioeconomic differentials in the utilization of these services were based on a limited number of factors, focusing either on preventive or on curative modern health services. These studies failed to collect data from remote rural areas of the different regions to examine the socioeconomic differentials in health-seeking behavior.

**Methods:**

Data from 3,498 randomly selected currently married women from three strata of households within 128 purposively chosen remote villages in three divisions of Bangladesh were collected in 2006. This study used bivariate and multivariate logistic analyses to examine both curative and preventive health-seeking behaviors in seven areas of maternal and child health care: antenatal care, postnatal care, child delivery care, mother's receipt of Vitamin A postpartum, newborn baby care, care during recent child fever/cough episodes, and maternal coverageby tetanus toxoid (TT).

**Results:**

A principal finding was that a household's relative poverty status, as reflected by wealth quintiles, was a major determinant in health-seeking behavior. Mothers in the highest wealth quintile were significantly more likely to use modern trained providers for antenatal care, birth attendance, post natal care and child health care than those in the poorest quintile (χ^2^, p < 0.01). The differentials were less pronounced for other factors examined, such as education, age, and the relative decision-making power of a woman, in both bivariate and multivariate analyses.

**Conclusion:**

Within rural areas of Bangladesh, where overall poverty is greater and access to health care more difficult, wealth differentials in utilization remain pronounced. Those programs with high international visibility and dedicated funding (e.g., Immunization and Vitamin A delivery) have higher overall prevalence and a more equitable distribution of beneficiaries than the use of modern trained providers for basic essential health care services. Implications of these findings and recommendations are provided.

## Background

In recent years, efforts to eliminate inequalities in the utilization of basic health care services have been emphasized for the overall improvement of health in developing countries [1-4]. As a part of efforts to provide basic preventive and curative health services to all, government and non-governmental organizations(NGOs) have been expanding their health services in rural Bangladesh. One purpose of this expansion was to make essential services available to all women and children [5-7]. Simultaneously, major efforts for improving the economic conditions of the poor have been occurring through massive microcredit programs throughout rural Bangladesh [5,8,9]. Increased income may also promote health by enabling the poor to purchase better health services. While some progress has been made in providing basic health services to poor women and their children, the progress may be uneven because many in the rural areas are difficult to reach with services [10-12].

Previous studies in rural Bangladesh have shown substantial socioeconomic inequalities in health status, access to health services, and their utilization, all disfavoring poor women and children [1,13-16]. There has been increasing availability and accessibility of practitioners of Western medicine in rural Bangladesh over the past decades [10-16]. While greater effectiveness of modern medicines in curing diseases may lead to the greater utilization of practitioners of western medicine compared to practitioners of traditional medicine, the utilization of the former is likely to be higher among those with higher socioeconomic status than among those with lower socioeconomic status [1,13-16]. Since the early 1980s there has been an increasing number of private facilities with modern Western medicines. Typically, their utilization is positively related to socioeconomic status [13-15].

A wide range of therapeutic choices in modern health care through public health facilities is available in rural Bangladesh. These include primary health care organized around the Health Complex located at the Upazila (sub-district) headquarters with in-patient and basic laboratory facilities. Attached to the Complex are two to three health sub-centers--Family Welfare Centers (FWCs)--at the Union (sub-divisions of an Upazila) level [7,10]. In Bangladesh there are 475 Upazilas within 64 districts within 6 Divisions. Eight to ten qualified allopathic practitioners and their auxiliary personnel staff an Upazila Health Complex, while para-professionals (a paramedic, a medical assistant, and a midwife) staff the union level sub-centers. These facilities provide a free essential services package (ESP) in health care, which consists of maternal health, family planning, communicable disease control, child health, and basic curative care [6,7,17]. Rural Bangladesh also has both drug stores selling traditional medicines and traditional practitioners of indigenous medicines [13,18,19].

Many factors limit the utilization of maternal and child health services in the rural areas of developing countries. These factors include the availability, accessibility, and quality of services as well as the characteristics of the users and communities in which the users live. Specifically, these may include distance to health service, cost of services, technical qualifications of health practitioners, socioeconomic status of the users, and women's autonomy in household decision-making [20-23]. Studies from rural Bangladesh found that some of these factors were positively associated with the utilization of health services [13-15,18,19]. However, many of these studies were based on a limited number of factors and focused on either preventive or curative modern health care services in small geographical areas [13-15,18,19,23-26]. As a result, they neither examined the net effects of a wider set of individual, community, and provider-level factors nor did they cover rural areas from different regions to see how the recent increase in the availability of maternal and child health services was affecting utilization by different socioeconomic groups [13-19,23-26]. Given the recent expansion of basic facilities, there is a need to examine how different socioeconomic groups have been affected [7,10,11].

This study examined socioeconomic differentials in maternal and child health-seeking behavior in selected rural areas from 3 of the 6 divisions of Bangladesh. The remainder of this paper is organized as follows. First, a conceptual model of health-seeking behavior is presented and some hypotheses about socioeconomic differentials in health-seeking behaviors are formulated [27]. Then the study setting, data, and variables are described. Next, bivariate relationships between health-seeking behaviors and socioeconomic indicators are documented and the multivariate results are presented. Finally, in the discussion section, the study findings are summarized and their policy implications elaborated.

## Methods

### Conceptual Framework

**Figure 1:**
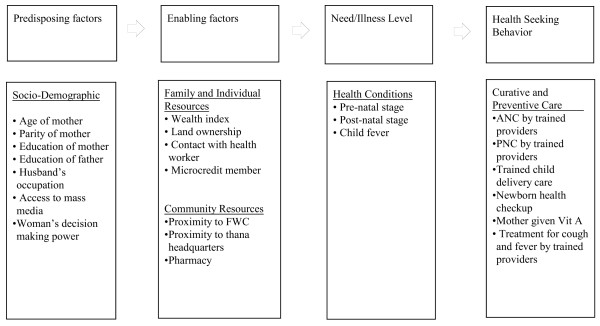
**Conceptual Framework for determinants of health seeking behaviors in rural Bangladesh**.

Figure 1 shows the conceptual model used in this study. In specifying the various factors influencing health-seeking behavior, we relied on a behavioral model and its subsequent modification [27-30]. The modified version of the model has been successfully applied in the study of health services utilization in developing countries [31,32]. This model proposes that health-seeking behavior is a function of three sets of individual characteristics: predisposing, enabling, and need. The actual seeking of health services is assumed to be a sequential and conditional function of the individual's predisposition to use health services, their perceived need to use them, and their ability to obtain the services. Some variables may belong in more than one of these categories. In such a case, we made an arbitrary classification for the analysis and presentation of our findings. The predisposing factors (i.e., age and parity of the mother, educational level of the father and mother, occupation of the father, exposure to mass media, and women's decision-making power), and enabling factors (i.e., wealth quintiles, pharmacy in the village, distance from a FWC, distance from Upazila headquarters, and microcredit group membership) are considered as independent variables affecting health-seeking behavior. The enabling factors are those by which individuals have the means that permit them to obtain health services. Finally, although predisposing and enabling factors are necessary for the use of health services, they are not sufficient for actual use; the actual use is triggered by the need during prenatal and postnatal stages, childbirth, or general illness [27]. In the present study, we explored the extent to which the predisposing and enabling factors contributed to any differences in health-seeking behavior.

### Hypotheses

We hypothesized that the seeking of basic curative and preventive health care services from modern trained providers by women in rural areas would be lower among mothers of lower socioeconomic strata than among those of higher socioeconomic strata. We also posited that this would be due to more physical, socioeconomic, and biomedical (services) constraints faced by the former than by the latter. Physical barriers to accessing health care in rural areas include fewer modern health services, lack of transportation and costs of transport. Therefore, women and children from lower socioeconomic strata are more likely to lag behind those from the higher socioeconomic strata in the utilization of services. Similarly, those from lower socioeconomic strata are likely to be more traditionally oriented about how to deal with their adverse health conditions as well as lacking knowledge about the causes, prevention and cures of illnesses. Those from lower socioeconomic strata are also more likely to encounter other constraints, such as apathy from health care providers and corrupt practitioners, which inhibit their access to and utilization of services. In contrast, those from the higher socioeconomic strata are more likely to have knowledge of and access to the services.

### The study setting, data, and variables

The data for this study came from a household survey carried out in 128 villages in 3 of the 6 Divisions of Bangladesh: Chittagong, Dhaka and Rajshahi. The survey included 3,498 currently married women residing in 3998 households in 128 villages outside 16 catchment areas of health centers of the Grameen Bank Health Program. This sample survey was a "baseline survey" for an experimental project with a 4-cell design to assess the relative effects of separately and jointly introducing additional microcredit and essential health services interventions on the use of health services, economic well-being, and women's empowerment.

Over the past decade the Grameen Health Program has established its non-governmental health centers in selected rural areas near the Grameen Banks in rural Bangladesh. Our study villages were located outside the catchment areas of these centers (more than 4-6 km from the center) and thus could be considered remote from those centers. Of the 31 Grameen centers located in 31 Upazilas (sub-districts) from three regions of Bangladesh, 16 centers in Upazilas with the lowest reported coverage of microcredit were first selected. An enumeration was then undertaken of 24 villages in the vicinity outside the catchment areas of the selected Grameen centers to find villages estimated to have less than 40-50% of households participating in microcredit and with only governmental health programs. On the basis of these results, villages with the lowest microcredit participation were selected from enumerated villages around the 16 centers. For each of the centers, two sets of four villages were selected in opposite directions from the center. None of the villages had NGO health services.

Prior to the household sample survey, a census was conducted in all 128 (16*8) villages. The purpose of this census was to categorize the households into three strata:

1) Not eligible for microcredit

2) Eligible and had accessed microcredit

3) Eligible but not accessed microcredit

Then in each village a stratified random sample was taken within these three strata among all households that had ever-married women. The sample sizes chosen were: 4, 12 and 15 from strata 1, 2 and 3 respectively. From the sample and census information, sampling weights were derived for each household and woman, and used in the analyses so results are representative of the 128 villages. The household response rate was 91.3% (3998 completed of 4381 sampled) and the eligible woman response rate was 98.7%.

Approval was obtained from the Institutional Review Boards of the Johns Hopkins School of Public Health and the Bangladesh Medical Research Council. The survey was conducted by a professional survey agency using a structured and pre-tested questionnaire. Thirty interviewers and supervisors (social science graduates who were experienced in survey methods) were recruited. They received training on the content of the questionnaires and techniques to elicit valid information by establishing rapport with the respondents while maintaining the neutrality essential to obtain the most accurate data possible. The training consisted of classroom lectures, role-playing, and practice sessions. Informed consent was obtained prior to conducting an interview.

Household and community information was collected from the heads of the households and community leaders, respectively. The woman's questionnaire included a birth history, details about maternal and child health care, recent childhood illness, microcredit membership, and relevant socioeconomic data. The survey was undertaken prior to the introduction of any intervention activities in the experimental areas of the project. More detailed information about the survey and its design is available elsewhere [5].

We examined the health-seeking behavior of the mothers in terms of their reproductive health care as well as the health care for their children who were born between June 2003 and September 2006. Only information for last-born children was examined. This restricted the analysis to births close to the time of the interview, and thus enhanced the likelihood that mothers provided accurate information about the reported use of health services. In view of the difficulty of separate care given to twins, the study focused on the 1261 singleton births available for analysis. Information on recent illness of the child and information on mother's reproductive health care was collected from the mothers. The health-seeking behaviors analyzed here are:

Antenatal and delivery care outcomes

(i) Trained antenatal care (ANC) provider vs. untrained provider or no ANC

(ii) Tetanus toxoid (TT) given vs. not given to the woman during the last live birth pregnancy

(iii) Child delivery by trained providers vs. untrained providers

Postpartum and child health outcomes

(iv) Trained postnatal care (PNC) provider vs. untrained provider or no PNC

(v) Newborn health checkup vs. no checkup

(vi) The mother's receipt of vitamin A within two months postpartum vs. no receipt

(vii) Trained provider vs. untrained or no provider for fever/cough of a child during a 15-day recall period prior to the interview.

The socioeconomic predictors of health-seeking behaviors consisted of the following: mother's level of schooling and father's occupation and level of schooling, membership in a microcredit group, and ownership of assets. Occupations were grouped into three categories: 1) agriculture; 2) skilled laborers (small businessmen, large businessmen, handicrafts, semi-skilled worker, low-level worker and middle-level worker) and 3) unskilled laborers (day laborers, poultry farming, rickshaw or cart puller, trawler or boat driver, unemployed, others and retired). Relative economic status of the households was determined through the creation of a wealth index. Wealth is assumed to be an underlying, theoretically measurable construct. It has been shown to be reliably assessed via a collection of indicators representing durable goods owned by the household, materials used in construction of the home, water and sanitation facilities and size of the home [33]. Instead of assigning equal weights to each of the indicators in the wealth index, principal components analysis was employed [34]. The analysis yields a factor score for each household. The assets used in the index were presence or absence of: electricity, a wardrobe, table, chair, clock, bed, radio, television, bicycle, at least one of motorcycle, sewing machine or telephone, brick, cement or tin walls, modern toilet or pit latrine. Also available was household density, measured as the number of people in a household divided by the number of rooms in the house. All but the last are binary indicators. The resulting asset scores for households were ordered and used to divide households into quintiles, representing their relative wealth with respect to other households in the study [1].

Other predictors of health-seeking behaviors examined in our analyses were age of the mother, her exposure to TV and/or radio, presence of a pharmacy in the village, distance to a family welfare centre (FWC), distance to Upazila headquarters, and women's decision-making power. Decision-making power was calculated from responses about each of 10 decision-making items. Specifically, each woman was asked: "In your family who do you think should have a say on decisions regarding: 1) buying costly furniture such as cot or showcase? 2) Whether to buy and to sell cows and goats? 3) How to spend family savings? 4) Whether to take a loan? 5) Treatment when a child is sick? 6) Whether to visit a doctor when you are sick? 7) Whether you can work for money outside the home? 8) Visiting your father's home? 9) Whether or not to have another child? 10) Whether or not to use family planning?" For each decision she was also asked: "Who takes part in the decision regarding the subject? "Among them, whose opinion is the most important on the decision regarding the subject?" and "Who has the final say on the decision regarding the subject?" For each item, we coded 0 if the woman reported that she did not participate in the decision, 1 if she reported that she contributed to the decision and 2 if she reported herself as the first or second most important person in actual decision-making. These values were then summed to provide the decision-making score used in the analyses. Previous studies have used similar variables in differentiating health care utilization in rural Bangladesh [13-15,17-19,23-26].

### Data Analysis

For each of the seven outcome variables, we calculated odds ratios with each covariate and conducted multivariate logistic regression analyses. For the bivariate analyses, we calculated significance using the χ^2 ^test of homogeneity. Independent variables were tested for multi-collinearity using the Pearson correlation coefficient and the variance inflation factor, and it was found not to be a problem (e.g. the highest correlation was 0.44 between years of schooling of spouses). Variables having an association of p < 0.1 in at least one of the bivariate analyses were included in all multivariate analyses. Additionally, age group of the mothers and wealth quintiles were included in all logistic regression analyses. For calculating the odds ratio for each category of the independent variables, the first group was always taken as the reference category. Analyses were done with STATA Version 9 with the SVY commands appropriate for sample surveys [35].

## Results

### Bivariate analyses

Bivariate results on socioeconomic differentials of preventive and curative health-seeking behaviors among the mothers are presented in Table 1. Greater use of antenatal care (ANC) from a trained provider was significantly associated with years of schooling of the mothers and the fathers, with 76.4% of mothers with more than primary school vs. 33.7% of mothers with no schooling seeking ANC from a trained provider (p < 0.01). Among women whose husbands had schooling above primary level 74.5% sought ANC from a trained provider compared with 35.9% of women whose husbands had no schooling (p < 0.01). Seeking of postnatal care, while overall less prevalent, also had significant differences by level of schooling of either parent. Similarly, mothers in families whose husbands were in agricultural or skilled labor occupations and whose households were in higher wealth quintiles were more likely to use modern providers for antenatal and postnatal care. Higher use of newborn health checkup and child delivery care by trained providers was also observed among those with more years of schooling and in higher wealth quintiles compared to those with less or no schooling and in lower wealth quintiles, respectively. Regarding the treatment of the children suffering from fever or cough, only 19.3% of the mothers used medically trained providers and none of the covariates had a significant effect. Overall, in Table 1, the differentials in the use of services from trained providers tended to be similar for both preventive and curative health care-seeking behaviors: those with lower socioeconomic status or in lower wealth quintiles were less likely to seek modern health care services than those with higher socioeconomic status or in higher wealth quintiles. This inequality was greatest between the highest and lowest quintiles. The use of TT (prevalence = 77.6%) and Vitamin A (prevalence = 23.7%) did not show any significant differences by wealth quintiles, or by education level of either parent. Interestingly, distance to FWC and to the Upazila Health complex (treated as continuous variables with a squared term to detect non-linear patterns) were not significant in any of the bivariate analyses, and were dropped from further analyses (not shown).

**Table 1:** Percent of women with given health seeking behaviors by selected socio-economic characteristics

		**Mother Care-seeking**						
				
		**Antenatal and Delivery Care**			**Postnatal Care**		**Child Care-seeking**	
				
**Indicator**		**ANC from trained provider**	**TT to mother during pregnancy**	**Attended by trained providers**	**PNC from trained provider**	**Mother given Vit A postpartum**	**Sought newborn checkup**	**Received treatment by trained providers for fever/cough**
Whole sample Wealth Quintile		56.9	77.6	32.2	23.1	23.7	26.3	19.3
	Low	31.5***	83.1	13.3***	5.1***	15.8	8.9***	9.5
	2^nd^	40.9	73.9	24.9	12.8	24.1	18.0	23.4
	3^rd^	49.4	87.0	27.2	13.4	22.1	17.1	12.3
	4^th^	59.1	88.2	25.0	19.9	20.5	23.9	21.0
	High	87.1	64.3	57.1	49.3	31.4	50.0	24.8
Membership in microcredit agency								
	Yes	48.7	81.4	23.4***	17.0**	23.9	20.1**	19.0
	No	57.7	80.4	39.0	29.3	23.9	32.9	21.5
Mother's Education								
	None	33.7***	81.1	15.9***	9.6***	15.2	16.0	11.1
	Some Primary	45.7	71.0	27.0	14.7	28.4	26.2	29.9
	> Primary	76.4	79.4	44.5	35.4	25.8	32.2	18.1
Father's Education								
	None	35.9***	79.5	19.6**	10.7**	16.0	17.1	15.9
	Some Primary	54.7	90.9	36.2	26.8	39.2	27.0	22.2
	> Primary	74.5	68.8	39.2	30.0	20.2	32.9	19.9
Father's Occupation								
	Agriculture	61.9	76.2	35.8	21.3	30.9	9.6***	16.0
	Unskilled labor	49.8	70.3	21.1	12.9	19.4	18.6	17.9
	Skilled	61.3	83.4	39.1	30.5	25.5	35.3	21.0
								
Number of observations		1261	1261	1261	1261	1261	1261	822

Indicators of significance from the test of homogeneity are given next to first category of each variable

** significant at 5%; *** significant at 1%

### Multivariate Analysis

The bivariate analyses showed that relative wealth, as reflected in wealth quintiles, was positively associated with health- seeking behavior, as were other indicators of socioeconomic status. Since some of the bivariate relationships may be confounded by other variables, we also carried out multivariate logistic regression analyses. The results of these analyses (Tables 2, 3 and 4) were largely consistent with those of the bivariate analyses. Independent variables in all analyses were: wealth quintiles, microcredit membership of the mother, years of schooling of the mother and the father, occupational group of the father, media exposure of the mother, age group of the mother, total decision-making score of the mother, and whether the community of the respondent had a pharmacy.

**Table 2:** Estimated odds ratios (and 95% confidence intervals) from multiple logistic regressions of antenatal and delivery care on selected socioeconomic and other indicators

**Indicator**	**Trained ANC provider vs untrained provider or no ANC**		**TT given vs not given**		**Trained Birth Attendant vs untrained or no attendant**	
			
**Socio-economic indicators**						
Relative Wealth: Poorest (reference)						
Quintile 2	1.28	[0.73-2.22]	0.48**	[0.25-0.92]	2.58**	[1.12-5.91]
Quintile 3	1.59	[0.72-3.51]	0.81	[0.41-1.56]	2.04	[0.71-5.87]
Quintile 4	1.70	[0.70-4.11]	0.78	[0.28-2.20]	1.68	[0.45-6.19]
Quintile 5	7.61***	[2.21-26.16]	0.42*	[0.16-1.09]	10.99***	[2.67-45.19]
Credit group member	1.74**	[1.05-2.89]	0.95	[0.49-1.83]	1.03	[0.52-2.04]
Mother'r's Years of Schooling						
1-5 years	1.00	[0.56-1.78]	0.60*	[0.34-1.05]	1.35	[0.73-2.50]
more than 5 years	2.65***	[1.39-5.06]	3.61***	[1.43-9.08]	2.49**	[1.23-5.05]
Father'r's Years of Schooling						
1-5 years	1.18	[0.64-2.15]	2.35**	[1.12-4.94]	0.96	[0.45-2.03]
more than 5 years	1.22	[0.48-3.09]	0.44*	[0.19-1.04]	0.45*	[0.20-1.02]
Father's occupation: Agriculture (reference)						
Unskilled labor	0.89	[0.41-1.91]	1.32	[0.51-3.40]	1.52	[0.67-3.47]
Skilled occupation	0.70	[0.34-1.44]	2.10	[0.68-6.48]	1.49	[0.72-3.06]
Media Exposure: none (reference)						
TV or Radio	1.38	[0.77-2.49]	1.44	[0.76-2.70]	0.62	[0.29-1.35]
TV and Radio	1.15	[0.52-2.54]	1.18	[0.54-2.55]	1.32	[0.55-3.16]
**Other Indicators**						
Age: <20 (reference)						
20-39	0.76	[0.36-1.61]	1.06	[0.40-2.80]	2.65*	[0.96-7.37]
40 +	1.19	[0.26-5.56]	1.54	[0.37-6.37]	2.72	[0.50-14.79]
Total decision making score	0.96	[0.90-1.04]	1.10**	[1.02-1.18]	0.91***	[0.86-0.97]
Pharmacy in village	1.28	[0.58-2.83]	0.57*	[0.29-1.10]	1.53	[0.80-2.93]
						
Number of observations	1212		1212		1212	

* significant at 10%; ** significant at 5%; *** significant at 1%

**Table 3:** Estimated odds ratios (and 95% confidence intervals) of postpartum care by selected socioeconomic and other indicators

**Indicator**	**Trained PNC provider vs untrained provider or no PNC**		**Mother received Vit A postpartum vs no Vit A**	
		
**Socio-economic indicators**				
Relative Wealth: Poorest (reference)				
Quintile 2	3.36**	[1.20-9.39]	1.53	[0.56-4.14]
Quintile 3	2.58	[0.73-9.06]	1.15	[0.38-3.54]
Quintile 4	7.42**	[1.61-34.29]	0.85	[0.24-3.01]
Quintile 5	34.93***	[6.30-193.64]	3.69**	[1.00-13.55]
Credit group member	1.53	[0.64-3.67]	1.52	[0.77-3.01]
Mother's Education: none (reference)				
1-5 years	0.60	[0.25-1.42]	1.68	[0.74-3.84]
more than 5 years	2.14*	[0.93-4.93]	1.80	[0.64-5.06]
Father's Education: none (reference)				
1-5 years	0.94	[0.42-2.08]	2.64***	[1.29-5.38]
more than 5 years	0.34*	[0.11-1.04]	0.54	[0.18-1.60]
Father's occupation: Agriculture (reference)				
Unskilled labor	1.18	[0.40-3.52]	1.19	[0.41-3.41]
Skilled occupation	0.97	[0.36-2.65]	1.12	[0.33-3.74]
Media Exposure: none (reference)				
TV or Radio	1.41	[0.37-5.41]	1.73	[0.78-3.84]
TV and Radio	3.28*	[0.87-12.40]	1.19	[0.45-3.14]
**Other Indicators**				
Age: <20 (reference)				
20-39	2.63	[0.70-9.90]	1.42	[0.57-3.55]
40 +	0.94	[0.10-9.19]	0.38	[0.08-1.92]
Total decision making score	0.96	[0.89-1.04]	0.97	[0.89-1.06]
Pharmacy in village	2.17*	[0.86-5.48]	1.06	[0.49-2.28]
				
Observations	1212		1212	

* significant at 10%; ** significant at 5%; *** significant at 1%

**Table 4:** Estimated odds ratios (and 95% confidence intervals) from logistic regressions of child care on selected socioieconomic and other indicators

**Indicator**	**Newborn health checkup vs no checkup**		**Child treated by trained provider vs treated by untrained provider or not treated during recent fever/cough illness**	
		
**Socio-economic indicators**				
Relative Wealth: Poorest (reference)				
Quintile 2	2.40**	[1.08-5.33]	4.83***	[1.93-12.05]
Quintile 3	2.58**	[1.01-6.59]	3.18**	[1.19-8.49]
Quintile 4	5.59***	[1.83-17.07]	7.36***	[1.95-27.86]
Quintile 5	21.33***	[5.77-78.88]	23.90***	[4.67-122.42]
Credit group member	1.04	[0.54-2.01]	1.37	[0.68-2.78]
Mother's Education: none (reference)				
1-5 years	0.93	[0.44-2.00]	3.24**	[1.30-8.10]
more than 5 years	0.89	[0.35-2.24]	2.35	[0.76-7.25]
Father's Education: none (reference)				
1-5 years	0.70	[0.38-1.29]	1.27	[0.52-3.13]
more than 5 years	0.53	[0.22-1.26]	0.77	[0.24-2.49]
Father's occupation: Agriculture (reference)				
Unskilled labor	5.49*	[0.96-31.35]	2.15	[0.50-9.22]
Skilled occupation	4.86*	[0.96-24.62]	2.04	[0.48-8.75]
Media Exposure: none (reference)				
TV or Radio	0.99	[0.37-2.64]	0.14***	[0.05-0.41]
TV and Radio	1.56	[0.58-4.19]	0.10***	[0.03-0.33]
**Other Indicators**				
Age: <20 (reference)				
20-39	1.39	[0.49-3.92]	1.27	[0.40-4.06]
40 +	0.63	[0.10-4.02]	0.13*	[0.01-1.16]
Total decision making score	0.97	[0.90-1.05]	1.17***	[1.06-1.29]
Pharmacy in village	1.72	[0.67-4.38]	0.56	[0.26-1.20]
				
Observations	1212		788	

* significant at 10%; ** significant at 5%; *** significant at 1%

Table 2 shows the adjusted odds of seeking ANC from a trained provider (of modern health care), receiving TT injection during pregnancy, and having a trained provider present at the time of childbirth, as compared to no or a non-trained provider or not having the TT injection. For each of these outcomes, the mother's years of schooling were significantly and positively associated with the desired behavior, ranging from 2.5 to 3.6 times greater odds for mothers with more than 5 years of schooling, compared to mothers with no formal education. Compared to the mothers in the lowest wealth quintile, mothers from the highest quintile had greater odds of seeking ANC from a trained provider (OR = 7.6, 95% CI: 2.2-26.2, p < 0.01) and nearly 11 times higher odds to have a trained provider present at childbirth (95% CI: 2.-45.2, p < 0.01). Schooling of the father, his occupation, and mother's media exposure did not demonstrate consistent significant associations with the three outcomes.

Post-partum care outcomes are shown in Table 3. Relative wealth, as reflected in wealth quintiles, was associated with the seeking of postnatal care (PNC) from a trained provider and receiving vitamin A within 2 months of delivery, with the strongest associations found when comparing the mothers in the highest quintile with those in the lowest quintile with adjusted odds ratios ranging from 3.7 to 34.9. A mother with more than primary education had a significantly greater chance of seeking PNC from a trained provider (OR = 2.1, 95% CI: 0.93-4.9, p < 0.10). However, when a father had greater than primary schooling, a mother was 66% *less *likely to seek PNC (p < 0.10).

Child related outcomes (Table 4) showed a striking association between wealth quintiles and care seeking, with those in higher wealth quintiles significantly more likely to seek newborn care or sick child care from a trained provider than those in the lowest quintile. Mothers with any schooling showed a positive pattern in seeking care from trained providers when a child was sick with fever or cough while babies whose fathers were unskilled laborers or skilled laborers were more likely to receive a newborn check-up. Mothers with any media exposure had 90% *lower *adjusted odds of seeking a trained provider for their sick child (OR = 0.13, 95% CI: 0.05, 0.37), when compared with those with no media exposure and, as expected, the mothers with greater decision-making power within the household were significantly more likely to seek trained care (p < 0.01).

## Discussion

In this study we examined socioeconomic differentials in maternal and child health care-seeking behavior among mothers in selected rural areas of Bangladesh by using a model encompassing predisposing and enabling factors. Both types of factors were significantly associated with socioeconomic differences in the utilization of maternal and child health services. One differential stood out-the inequality in the utilization of services by wealth quintiles was large and statistically significant. This evidence is consistent with that of other studies [14,15,18,19].

Socioeconomic differentials in the utilization of curative health services for fever and cough, preventive health services of ANC and PNC, and delivery care were more pronounced than those of TT immunization of pregnant mothers and vitamin A supplementation. TT has been a part of a massive Expanded Program of Immunization (EPI) campaign in rural Bangladesh since the early 1980s, so its coverage has become high throughout the country. This wider coverage of EPI could be due to the fact that those below the poverty line are reached by the special efforts of highly subsidized EPI services, which are cost-effective and can reach the doorsteps of households in remote areas via mobile units [12,36-38]. Similarly, the relatively small differentials in vitamin A supplementation by socioeconomic status could be due to its free distribution under the government's $60 million nutritional program, which reduced disparities by targeting those from low-income households [10,39]. Because maternal and child health services in the curative area of fever treatment and preventive areas of prenatal, delivery, and postnatal care are neither available as widely nor accessible as equitably as TT, their overall use was lower and their use by those in lower wealth quintiles was low compared to that of those in higher quintiles. Results such as the negative association between media exposure and seeking trained providers for a child's fever/cough or the negative association between a father's years of schooling and PNC, are more puzzling. It is possible that women with greater media exposure feel more confident in providing self-care for their sick children, or that women without media exposure are differentially misreporting the source of care for their children, through some social desirability bias. With regard to predicted lower PNC use when husbands had more schooling in the multivariate model, despite a VIF value of only 0.4, it appears that some undetected multicollinearity is causing this relationship. When we regressed seeking PNC on the same covariates minus the woman's schooling and media variables, the relationship between father's schooling and PNC became non-significant. Despite these caveats, results show that a stark inequality in the utilization of modern maternal and child health services persists in rural Bangladesh.

A limitation is that these data were from a purposive sample of villages in relatively remote rural areas. Therefore, the findings pertain to the population of households of the 128 sampled villages at time of the interview, strictly speaking. Despite this, our findings are similar to those found in previous studies, both from purposely and randomly selected villages [1,6,12-16,19,24-26].

## Conclusion

On the basis of the findings of this research, we suggest that specific efforts are needed to target women and children of lower socio-economic status (especially, those within the lower wealth quintiles), with basic maternal and child health care services. Both formal education and relative wealth were positively associated with the utilization of maternal and child health services. Consequently, both the economic and educational improvement of the poor mothers would have a reinforcing effect on improved service utilization, so they both need to be strengthened.

In recommending the above, we recognize that the marginal cost of providing health services to the poor in rural areas is more than the average cost in any population. This is due to the inability of the poor to pay user fees and the high cost of reaching them with effective services [6,37]. This study showed that microcredit participation was positively associated with the use of trained providers of ANC. Poor mothers are likely to contribute to household resources through their microcredit participation, and thus increase the household's ability to pay for health services. Nevertheless, there are other barriers such as lack of access to, and information regarding, quality health services, that may inhibit their use of the services. Since an increasing number of poor women are taking microcredit loans and their participation could enhance their decision-making power with regard to health services utilization, strengthening of microcredit programs may also indirectly promote utilization of health services [8,40,41]. In addition, microcredit-linked health insurance programs can promote affordable health care and enhance access to quality care [42]. These pro-poor measures are likely to reduce inequality and inequity in access to, and utilization of, both preventive and curative health services. All these will enable Bangladesh to overcome barriers to the attainment of Millennium Development Goals by the year 2015 [43].

## List of abbreviations used

ANC: Antenatal care; DHS: Demographic and Health Survey; EPI: Expanded Program of Immunization; FWC: Family Welfare Center; PNC: Postnatal care; TT: Tetanus Toxoid.

## Competing interests

The authors declare that they have no competing interests.

## Authors' contributions

RA conceived of the study, developed its design, prepared the initial draft of the manuscript, and revised it as per anonymous reviewers' comments. NS performed the statistical analyses and participated in the drafting and revision of the manuscript. SB is PI of the larger study, led its design and coordinated data collection, advised on statistical analyses, and assisted in revision of the manuscript. All authors read and approved the final manuscript.

## References

[B1] Gwatkin DR Rustein S Johnson K Suliman E Wagstaff A Amouzou A Socioeconomic differences in health, nutrition and population in Bangladesh 2000 Washington, DC, The World Bank http://www.WorldBank.org/hnppublications

[B2] Gwatkin DR Bhuiya A Victora CG Making health more equitable Lancet 2004 364 1273 1280 10.1016/S0140-6736(04)17145-6 15464189

[B3] Victora CG Wagstaff A Schellenberg JA Gwatkin DR Claeson M Habicht JP Applying equity lens to child health and mortality: More of the same is not enough Lancet 2002 362 233 241 10.1016/S0140-6736(03)13917-7 12885488

[B4] Wagstaff A Poverty and Health. CMH Working Paper Series 2001 WG1: 5 Washington DC, The World Bank http://www.cmhealth.org/wg1_Paper5.pdf

[B5] Amin R Grameen Microcredit to Grameen Kalyan health programme for the poor: Reasons for optimism 2007 Academic Press and Publishers: Dhaka, Bangladesh

[B6] Ensor T Dave-Sen P Ali L Hossain A Begum SA Moral H Do essential service packages benefit the poor? Preliminary evidence from Bangladesh Health Policy and Planning 2002 17 247 256 10.1093/heapol/17.3.247 12135990

[B7] Government of Bangladesh Health, nutrition and population strategic investment plan: July 2003-June 2006 2004 Ministry of Heath and Family Welfare, Dhaka, Bangladesh

[B8] Zaman H The scaling-up of microfinance in Bangladesh: Determinants, impact, and lesson. World Bank Policy Research Working Paper Series No 3398 2004 Washington, DC, The World Bank full_text

[B9] Yunus M Grameen Bank, microcredit, and millennium development goals Economic and Political Weekly 2004 39 4077 4080

[B10] Jahan R Securing maternal health through comprehensive reproductive health services: lessons from Bangladesh American Journal of Public Health 2007 97 1186 1190 10.2105/AJPH.2005.081737 17538067 PMC1913082

[B11] Perry H Health for all in Bangladesh: Lessons in Primary Health Care for the Twenty-First Century 2000 Dhaka, Bangladesh: The University Press Limited

[B12] National Institute of Population Research and Training (NIPORT), Mitra and Associates, Macro ORC Bangladesh Demographic and Health Survey 2004 2005 Dhaka, Bangladesh and Calverton, Maryland, USA

[B13] Ahmed SM Petzold M Kabir ZN Tomson G Targeted intervention for the ultra poor in rural Bangladesh: Does it make any difference in their healthseeking behavior? Social Science and Medicine 2006 63 2899 2911 10.1016/j.socscimed.2006.07.024 16954049

[B14] Anwar ATMI Killewo J Chowdhury ME Dasgupta SK Bangladesh: Inequalities in utilization of maternal health care services - Evidence from Matlab 2004 Washington, DC, The World Bank http://www.WorldBank.org/hnppublications

[B15] Rahman MH Mosley WH Ahmed S Akhter HH Does service accessibility reduce socioeconomic differentials in maternal care seeking? Evidence from rural Bangladesh Journal of Bio-social Science 2007 40 19 33 10.1017/S0021932007002258 17588280

[B16] Koenig MA Jamil K Streatfield PK Saha T Ahmed AS Arifeen SE Hill K Haque Y Maternal health and care-seeking behavior in Bangladesh: Findings from a national survey International Family Planning Perspectives 2007 33 75 82 10.1363/3307507 17588851

[B17] Arifeen SE Bryce J Gouws E Baqui AH Black RE Hoque DM Quality of care for under-fives in first-level health facilities in one district of Bangladesh Bulletin of the World Health Organization 2005 83 260 267 15868016 PMC2626213

[B18] Amin R Chowdhury SA Kamal GM Chowdhury J Community health services and health care utilization in rural Bangladesh Social Science and Medicine 1989 29 1343 1349 10.1016/0277-9536(89)90234-7 2697945

[B19] Amin R St Pierre M Ahmed A Huq M Integration of an Essential Services Package (ESP) in Child and Reproductive Health and Family Planning with a Microcredit Program for Poor Women: Experience from a Pilot Project in Rural Bangladesh World Development 2001 29 1611 1621 10.1016/S0305-750X(01)00055-9

[B20] Caldwell JC Routes to low mortality in poor countries Population and Development Review 1986 12 171 220 10.2307/1973108 21174865

[B21] Cleland JG Van Ginneken JK Maternal education and child survival in developing countries: the search for pathways of influence Social Science and Medicine 1988 27 1357 1368 10.1016/0277-9536(88)90201-8 3070762

[B22] Das Gupta M Death clustering, mother's education and the determinants of child mortality in rural Punjab, India Population Studies 2004 44 489 505 10.1080/0032472031000144866

[B23] Levin A Rahman MA Quayyum Z Routh S Barkat-e-Khuda The demand for child curative care in two rural thanas of Bangladesh: effect of income and women's employment International Journal of Health Planning and Management 2001 16 179 194 10.1002/hpm.630 11596556

[B24] Paul BK Rumsey DJ Utilization of health facilities and trained birth attendants for childbirth in rural Bangladesh: an empirical study Social Science and Medicine 2002 54 1755 1765 10.1016/S0277-9536(01)00148-4 12113433

[B25] Ahmed SM Adams AM Chowdhury M Bhuiya A Gender, socioeconomic development and health-seeking behaviour in Bangladesh Social Science and Medicine 2000 51 361 371 10.1016/S0277-9536(99)00461-X 10855923

[B26] Ahmed SM Tomson G Petzold M Kabir ZN Socioeconomic status overrides age and gender in determining health-seeking behavior in rural Bangladesh Bulletin of the World Health Organization 2005 83 109 117 15744403 PMC2623805

[B27] Andersen RM A behavioral model of families' use of health services 1968 Chicago, Illinois, USA: Center for Health Administration Studies, University of Chicago

[B28] Aday LA Andersen RM A framework for the study of access to medical care Health Services Research 1974 9 208 220 4436074 PMC1071804

[B29] Andersen RM Newman JF Societal and individual determinants of medical care utilization in the United States Milbank Memorial Fund Quarterly 1973 51 95 124 10.2307/3349613 4198894

[B30] Andersen RM Revisiting the behavioral model and access to medical care: does it matter? Journal of Health and Social Behavior 1995 36 1 10 10.2307/2137284 7738325

[B31] Fosu GB Childhood morbidity and health services utilization: Cross-national comparisons of user-related factors from DHS data Social Science and Medicine 1994 38 1209 1220 10.1016/0277-9536(94)90186-4 8016686

[B32] Subedi J Modern health services and health care behavior: a survey in Kathmandu, Nepal Journal of Health and Social Behavior 1989 30 412 420 10.2307/2136989 2600384

[B33] Rutstein SO Johnson K The DHS wealth index. DHS comparative reports no. 6 2004 Calverton, MD. ORC Macro

[B34] Filmer D Pritchett LH Estimating wealth effects without expenditure data--or tears: an application to educational enrollments in states of India Demography 2001 38 115 132 11227840 10.1353/dem.2001.0003

[B35] StataCorp. Statistical Software Release 9.0 2005 College Station, TX: Stata Corporation

[B36] Huq M Near miracle in Bangladesh 1991 Dhaka, Bangladesh: University Press Limited

[B37] Ensor T Cooper S Overcoming barriers to health service access: influencing the demand side Health Policy and Planning 2004 19 69 79 10.1093/heapol/czh009 14982885

[B38] Amin R Li Y NGO-Promoted women's credit program, immunization coverage, and child mortality in rural Bangladesh Women and Health 1997 25 71 87 10.1300/J013v25n01_05 9253139

[B39] Hossain S Duffield AA Taylor A An evaluation of the impact of a $60 million nutrition program in Bangladesh Health Policy and planning 2005 20 35 40 10.1093/heapol/czi004 15689428

[B40] Amin R Becker S Bayes A NGO-promoted microcredit programs and women's empowerment in rural Bangladesh: quantitative and qualitative evidence Journal of Developing Areas 1998 32 221 236 12294125

[B41] Osmani LNK Impact of credit on the relative well-being of women: Evidence from Grameen Bank IDS BULLETIN 1998 29 30 38 10.1111/j.1759-5436.1998.mp29004004.x

[B42] Churchill C Churchill C What is insurance for the poor? Protecting the poor A microinsurance compendium 2006 Geneva: International Labour Organization (ILO) 12 24

[B43] United Nations Road map towards the implementation of United Nations Millennium Declaration: Report of the Secretary-General. New York, NY 2001

